# Supplementary material for: Non-Immersive Virtual Reality Telerehabilitation System Improves Postural Balance in People with Chronic Neurological Diseases
Source: J Clin Med. 2023 Apr 28;12(9):3178. doi: 10.3390/jcm12093178 (PMC10179507; doi:10.3390/jcm12093178)
Supplement: Supplementary file 1 [file jcm-12-03178-s001.zip › jcm-2236554-supplementary.pdf]

Table S1. Baseline demographics and clinical data in the TR and CG.

Group	Variables	People with MS	People with PD	Group comparison [p-value]
CG	**Age years, [M, (SD)]**	52.23 (9.34)	68.32 (5.89)	<.001§
	**Education, N (%)**			
	**Primary**	0 (0%)	3 (8.1%)	0.094^
	**Secondary**	5 (16.7%)	12 (32.4%)	
	**High School**	16 (53.3%)	17 (45.9%)	
	**College**	9 (30.0%)	5 (13.5%)	
	**Sex (Male/Female), N (%)**	12 (40%)/18 (60%)	18 (49%) /19 (51%)	.479^
	**Mini-BESTest, [M, (SD)]**	18.10 (6.23)	21.00 (5.52)	.048§
	**Mini-BESTest Anticipatory postural control item, [M, (SD)]**	3.77 (1.59)	4.05 (1.51)	.452§
	**Mini-BESTest Reactive postural control item, [M, (SD)]**	3.80 (2.12)	4.54 (1.64)	.112§
	**Mini-BESTest Somatosensory orientation item, [M, (SD)]**	4.07 (1.46)	5.00 (1.27)	.007§
	**Mini-BESTest Dynamic walking item, [M, (SD)]**	6.47 (2.56)	7.41 (1.95)	.093§
	**TUG [ln], [M, (SD)]**	2.51 (0.41)	2.17 (0.50)	.004§
	**TUG-D [ln], [M, (SD)]**	2.64 (0.44)	2.38 (0.55)	.033§
	**MoCA, [M, (SD)]**	25.73 (3.34)	24.76 (3.09)	0.302*
TG	**Age years, [M, (SD)]**	48.33 (9.66)	66.51 (7.37)	<.001§
	**Education, N (%)**			
	**Primary**	0 (0%)	0 (0%)	0.223^
	**Secondary**	4 (13.3%)	9 (25.7%)	
	**High School**	20 (66.7%)	16 (45.7%)	
	**College**	6 (20.0%)	10 (28.6%)	
	**Sex (Male/Female), N %**	12 (40%)/ 18 (60%)	17 (49%)/ 18 (51%)	.488^
	**Mini-BESTest, [M, (SD)]**	18.93 (5.98)	19.86 (5.60)	.523§
	**Mini-BESTest Anticipatory postural control item, [M, (SD)]**	3.63 (1.50)	4.20 (1.57)	.143§
	**Mini-BESTest Reactive postural control item, [M, (SD)]**	4.03 (2.08)	4.37 (1.63)	.465§
	**Mini-BESTest Somatosensory orientation item, [M, (SD)]**	4.37 (1.59)	4.71 (1.38)	.349§
	**Mini-BESTest Dynamic walking item, [M, (SD)]**	6.90 (2.14)	6.60 (1.99)	.560§
	**TUG [ln], [M, (SD)]**	2.45 (0.50)	2.15 (0.54)	.030§
	**TUG-D [ln], [M, (SD)]**	2.64 (0.55)	2.39 (0.58)	.069§
	**MoCA, [M, (SD)]**	26.30 (2.67)	25.51 (2.66)	0.774*

Legend: PD= Parkinson's Disease; MS= Multiple Sclerosis; CG=Control Group; TG=Telerehabilitation Group; M=mean; SD=standard deviation; Mini-BES Test= mini-Balance Evaluation Systems Test; TUG=Timed Up & Go; TUG-D= Timed Up-and-Go-test Dual-task ; MoCA=Montreal Cognitive Assessment; MDS-UPDRS= MDS-Unified Parkinson's Disease Rating Scale; PDQ-8=Parkinson's Disease Questionnaire; MSQOL-54 PHCS=Multiple Sclerosis Quality of Life-54 Pysical Health Composite Score; MSQOL-54_ MHCS=Multiple Sclerosis Quality of Life-54 Mental Health Composite Score. Statistical comparisons between TG and CG were performed using Independent sample t-test (§); ANCOVA adjusting for age and education (*); ANCOVA adjusting for age (#); Mann-Whitney U test (°); chi-square (^); ln=Logarithm Natural transformation was applied to account for no normal distribution. Statistically significant results (p-value<0.05).
